# Automated screening for Fragile X premutation carriers based on linguistic and cognitive computational phenotypes

**DOI:** 10.1038/s41598-017-02682-4

**Published:** 2017-06-01

**Authors:** Arezoo Movaghar, Marsha Mailick, Audra Sterling, Jan Greenberg, Krishanu Saha

**Affiliations:** 1 Waisman Center, University of Wisconsin-Madison, Madison, WI USA; 2 Wisconsin Institute for Discovery, University of Wisconsin-Madison, Madison, WI USA; 3 Department of Biomedical Engineering, University of Wisconsin-Madison, Madison, WI USA; 4 Department of Communication Sciences and Disorders, University of Wisconsin-Madison, Madison, WI USA; 5 Department of Social Work, University of Wisconsin-Madison, Madison, WI USA

## Abstract

Millions of people globally are at high risk for neurodegenerative disorders, infertility or having children with a disability as a result of the Fragile X (FX) premutation, a genetic abnormality in *FMR1* that is underdiagnosed. Despite the high prevalence of the FX premutation and its effect on public health and family planning, most FX premutation carriers are unaware of their condition. Since genetic testing for the premutation is resource intensive, it is not practical to screen individuals for FX premutation status using genetic testing. In a novel approach to phenotyping, we have utilized audio recordings and cognitive profiling assessed via self-administered questionnaires on 200 females. Machine-learning methods were developed to discriminate FX premutation carriers from mothers of children with autism spectrum disorders, the comparison group. By using a random forest classifier, FX premutation carriers could be identified in an automated fashion with high precision and recall (0.81 F1 score). Linguistic and cognitive phenotypes that were highly associated with FX premutation carriers were high language dysfluency, poor ability to organize material, and low self-monitoring. Our framework sets the foundation for computational phenotyping strategies to pre-screen large populations for this genetic variant with nominal costs.

## Introduction

The fragile X mental retardation 1 (*FMR1*) gene is associated with a wide spectrum of clinical involvement. *FMR1* is responsible for coding a protein called the fragile X mental retardation protein (*FMRP*), which plays a key role in healthy brain development and functioning. The absence or reduction of this protein has been linked with cognitive deficits^1–4^. Abnormalities in the *FMR1* gene occur when there is a cytosine-guanine-guanine (CGG) repeat expansion on the X chromosome. This expansion results in *FMR1* transcriptional silencing, which decreases the amount of *FMRP* ^1–4^. Elevated CGG repeats beyond 55 signify either the FX premutation (55–200 repeats) or the full mutation (>200 repeats). The full mutation of the *FMR1* gene results in Fragile X syndrome (FXS), which is the most common inherited cause of intellectual disability, and affects approximately one in every 2500 males and one in every 6000 females^1–3, 5, 6^. This syndrome causes significant impairments in intellectual and language development.

While the full mutation of FXS is relatively rare, the FX premutation is much more common. A population-based US study of the prevalence found that 1 in every 151 females and 1 in 468 males had the FX premutation^7^, thus affecting approximately one million Americans. Repeats can expand in number as they are transmitted from generation to generation. In women, the FX premutation can expand to the full mutation in oocytes. Therefore, female FX premutation carriers have an increased risk of having a child with the full mutation of FXS^8–13^.

Originally FX premutation carriers were believed to be clinically unaffected. However, recent research has uncovered a range of clinical involvement including a neurodegenerative disorder (i.e., fragile X-associated tremor ataxia syndrome, FXTAS) that impacts about 40% of male FX premutation carriers and 8% of female FX premutation carriers over the age of 50^4^. For those FX premutation carriers who do not develop FXTAS, there are a significant number of individuals who develop an aging related set of cognitive symptoms, including impairments in working memory and executive function skills (e.g., deficits in planning and organization)^8–12^. Despite growing evidence of the cognitive dysfunction in females with the FX premutation and high prevalence of the premutation in females, the majority of past research has focused on male FX premutation carriers. Therefore, there is a critical need for understanding the cognitive phenotype in female FX premutation carriers^14^.

**Figure 1:**
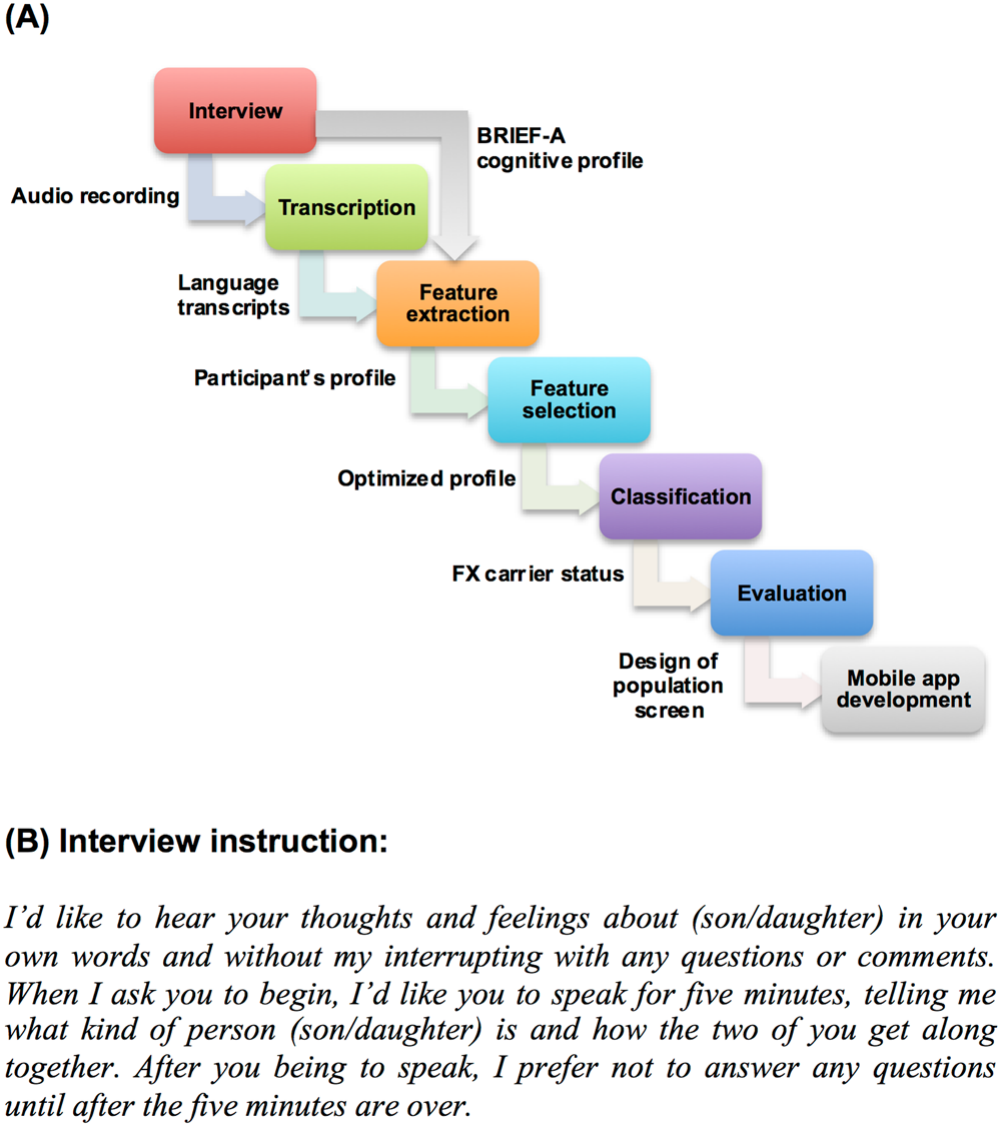
Workflow overview and language sample instruction. (**A**) The workflow for this project starts with data collection via phone or in-person interviews. Five-minute language samples were generated in response to interview instruction shown in (**B**) as previously described^11^. The audio recordings were transcribed and a text-processing module was used to extract linguistic features from the resulting transcripts. In parallel, cognitive profiles were collected using a cognitive self-assessment based on BRIEF-A standard^32, 33^. Then, a comprehensive profile was generated using the combination of linguistic and cognitive features. For the machine learning portion of the study, a feature selection module was implemented to choose the most informative features based on the information gain score. A classifier was developed to determine the FX premutation status. Finally, the results of evaluation were used to determine the feasibility of improving data collection process. A mobile app based on these results was developed to expand the study.

FX premutation carriers are often undiagnosed and are only identified if they have a child with the full mutation, or a history of FXS in the family. Because genotyping for elevated repeats is resource intensive, it would be advantageous to develop a method to rapidly screen individuals for FX premutation status. Our team has collected data on FXS-affected families through several research projects. The main focus of our first study^13^ was to examine the impact of FXS on families, particularly the impact on the FX premutation carrier mothers of their adolescent and adult children with FXS. In this longitudinal study, multiple interviews with family members have been performed and various types of phenotypic data were collected from the volunteer families living in 38 states. In addition, a five-minute language sample was collected, which is the primary source of data analyzed for the present study. The language sample was provided in response to the instruction in Fig. 1 ^15^.

Among the studies that have emerged from this project, Greenberg *et al*.^17^ used the language samples to measure the female FX premutation carriers’ expressed emotion (EE), which refers to the emotional environment of the family^16^. This research was the first to examine the relationship between EE and behavior problems in individuals with FXS across different stages of the child’s life course, and found that the presence of parental warmth and positivity and the absence of parental criticism (measured via language samples) were associated with fewer behavior problems in the son or daughter with FXS^13, 17^. Previously, this group used the five minute language samples to rate mothers’ expression of EE toward their adolescent or adult son or daughter with autism spectrum disorder^18^.

Sterling *et al*. (2013) reanalyzed these same language samples, this time examining language dysfluency as one aspect of potential neurocognitive decline in FX premutation carriers. Past research has demonstrated that language dysfluencies are one indicator of executive function deficits^19^. Dysfluencies are a hallmark feature of other neurocognitive disorders, including Parkinson’s disease^20^, and Alzheimer’s disease^21^. This study was the first examination of the language phenotype in FX premutation adult female carriers. The results indicated a strong positive correlation between age and dysfluency in FX premutation carriers, suggesting that dysfluency could be an indicator of cognitive decline in this population^11^.

In the present study, we sought to investigate the use of computational phenotyping to identify FX premutation carriers from a high dimensional cognitive and linguistic profile without relying on genetic data, and to develop easy and cost effective methods to prescreen the population for FX premutation status. In the proposed framework we improve the accuracy of the classification process by using machine-learning methods. By considering the possibility of using new technologies such as mobile devices, we improve the efficiency of the data collection process and provide access to pre-screening tools for this genetic variant in the broader population.

A group of 100 FX premutation carrier mothers of adolescent and adult children with full mutation FXS were compared to an age- and education-matched comparison group of 100 mothers of individuals with autism spectrum disorder (ASD). We analyzed the five-minute language samples that had previously been collected^13, 17^ during 200 interviews with these study participants. From the language samples, 88 linguistic features were extracted and over 12,850 sentences were analyzed. The resulting profile is extensive in terms of the diversity of features and intensive in terms of detailed definition of each feature. Integrating cognitive and linguistic features grows the phenotypic space of our study significantly. Due to the temporal nature and context dependency of cognitive features in general, they add new challenges and dimension to our phenotypic space^22^. Therefore, traditional statistical methods that build relationships between limited number of inputs and one single outcome are poorly suited to analyzing this large amount of data with high dimensionality.

Building on prior approaches that analyze data among multiple genotypes and multiple phenotypes^22^, we used statistical and machine-learning methods to develop a feature selection module and a data-driven classifier (Fig. 1). Through a phenotype discovery process, we found that the highest ranked features come from linguistic profiling including distribution, complexity, density of language and dysfluency patterns. Adding executive function features such as self-reported difficulties with organization of material and self-monitoring to the feature set improved the efficiency of the final classifier. Notably, these features potentially can be measured using an app on mobile devices through an easy, user-friendly process involving answering simple questions and a brief five-minute audio recording. Incorporating our novel system with mobile devices provides new opportunities such as global data collection, scaling up research studies, and tracking and monitoring cognitive changes in the participants. An efficient computational approach built on data easily obtained from individuals could optimize the use of resources to rapidly identify FX premutation carriers^14, 22^.

## Results

### Participants

FX premutation carriers were part of a national study, Family Adaptation to FXS^11, 13, 17^. This multisite study included 147 families from 38 states verified to have a son/daughter, age 12 or older, with the full mutation of the *FMR1* gene causing FXS. Premutation status of the mothers in these families was verified from medical records or genetic testing. The mean CGG repeat length was 98 (SD = 20.4). This dataset has a over-representation of individuals with longer CGG repeats, when compared to the distribution of CGG repeat length observed in the general US population^23^. The main aim of this study was to examine the impact of FXS on families as well as the impact of the family environment on the individual with FXS (more details are in refs 17, 18).

For the comparison group, mothers of individuals with ASD were drawn from the Adolescents and Adults with Autism study, conducted in Wisconsin and Massachusetts^24^. The study had a total of 406 participants (202 families living in Wisconsin and 204 families in Massachusetts). All of these families had a son or daughter older than age 10 when the study began, and had an independent diagnosis of ASD (more details on methodology and findings are in refs 17, 24–29).

For both studies, written informed consent was obtained prior to testing. The institutional review board at University of Wisconsin- Madison approved the studies. Linguistic and cognitive information were collected via interviews (in-person and telephone) and self-administered questionnaires in order to create a personalized profile for each participant. No significant differences (*p* > 0.05) were observed between language sample features measured via phone or in-person interviews (see Supplementary Table S6 and Supplementary Text). Therefore, consistent with previous reports^30, 31^, the measures used in this study can be collected either in person or over the phone.

For the present study, we selected 200 sample members (100 FX premutation carriers who were mothers of individuals with the full mutation of FXS and 100 mothers of individuals with ASD in comparison group). The selection process was based on availability of both cognitive and linguistic profiles as well as age and education matching (Table 1).

**Table 1:** Participants’ information and results of independent two-sample t-test.

	FX premutation carriers		Comparison group		*t*	*p*
	Mean	SD	Mean	SD		
Maternal age	48.91	6.332	47.374	6.886	1.633	0.104
Maternal education	3.16	0.66	3.14	0.75	0.201	0.841

Demographic information and comparison for all participants in this study. Maternal education scaled as follows: 1: Less than high school; 2: High school graduate; 3: Some college/Bachelor’s degree; and, 4 Post bachelor’s/grad degree.

### Measures and Features

Cognitive profiles were created based on scores from the Behavior Rating Inventory of Executive Function Adult Version (BRIEF-A)^32–35^. The BRIEF-A is a standardized self-report measure assessing executive functioning and the ability to self-regulate in adults. Linguistic profiles were extracted from extensive phenotyping of the five-minute language samples collected during the interviews. Definitions and examples of the linguistic features are provided in Table 2 (for more details, see Supplementary Table S5).

**Table 2:** Valuable linguistic and cognitive features.

Type		Features	Description	Utterance example
*Linguistic profile*	*Standard Dysfluency*	Filled pauses	Words or vocalizations that fill in a pause, e.g., um, ah, oh.	She is uh a very uh lovely girl.
		Total number of Repetitions	The exact duplication of a linguistic unit of any length, from a word to an entire clause with no other utterances besides fillers (such as “um” or “uh” allowed in between	
		Repetition (word)	Number of repetitions of single words	(He) He is John.
		Repetition (phrase)	Number of repetitions at the phrase level	(He is) He is John.
	*Complexity and dysfluency pattern*	Number of utterances	Number of all the spoken statements, questions, exclamations, or vocal sound. An utterance is any independent clause and any dependent clauses or phrases associated with it.	What is his name? I don’t know! He is John.
		Number of statements	Number of all the statements (utterances ending with “.”)	He is John.
		Number of questions	Number of all the questions (utterances ending with “?”)	What is his name?
		Mean length of utterances	Average number of morphemes per utterance	
		One-word utterance	Number of utterances, which their length is equal to 1.	Yes.
		Number of repeated words	Total number of words occurring in a dysfluency.	
		Repeated words Percentage	Rate of the repeated words per all words produced by speaker.	
		Short utterances	Utterances with less than 5 morphemes	
		Medium utterances	Utterances with 6 to 10 morphemes	
		Average repetitions per utterance	Average number of repetitions occurred per utterances	
*Cognitive profile*		Self-monitoring	The Self-Monitor scale from BRIEF-A assesses aspects of social or interpersonal awareness. It captures the degree to which an individual perceives himself as aware of the effect that his or her behavior has on others.	
		Organization of material	The Organization of Materials scale from BRIEF-A measures orderliness of work, living, and storage spaces.	
		Working memory	The capacity to hold information in mind for the purpose of completing a task, encoding information, or generating goals, plans, and sequential steps to achieving goals.	

**Figure 2:**
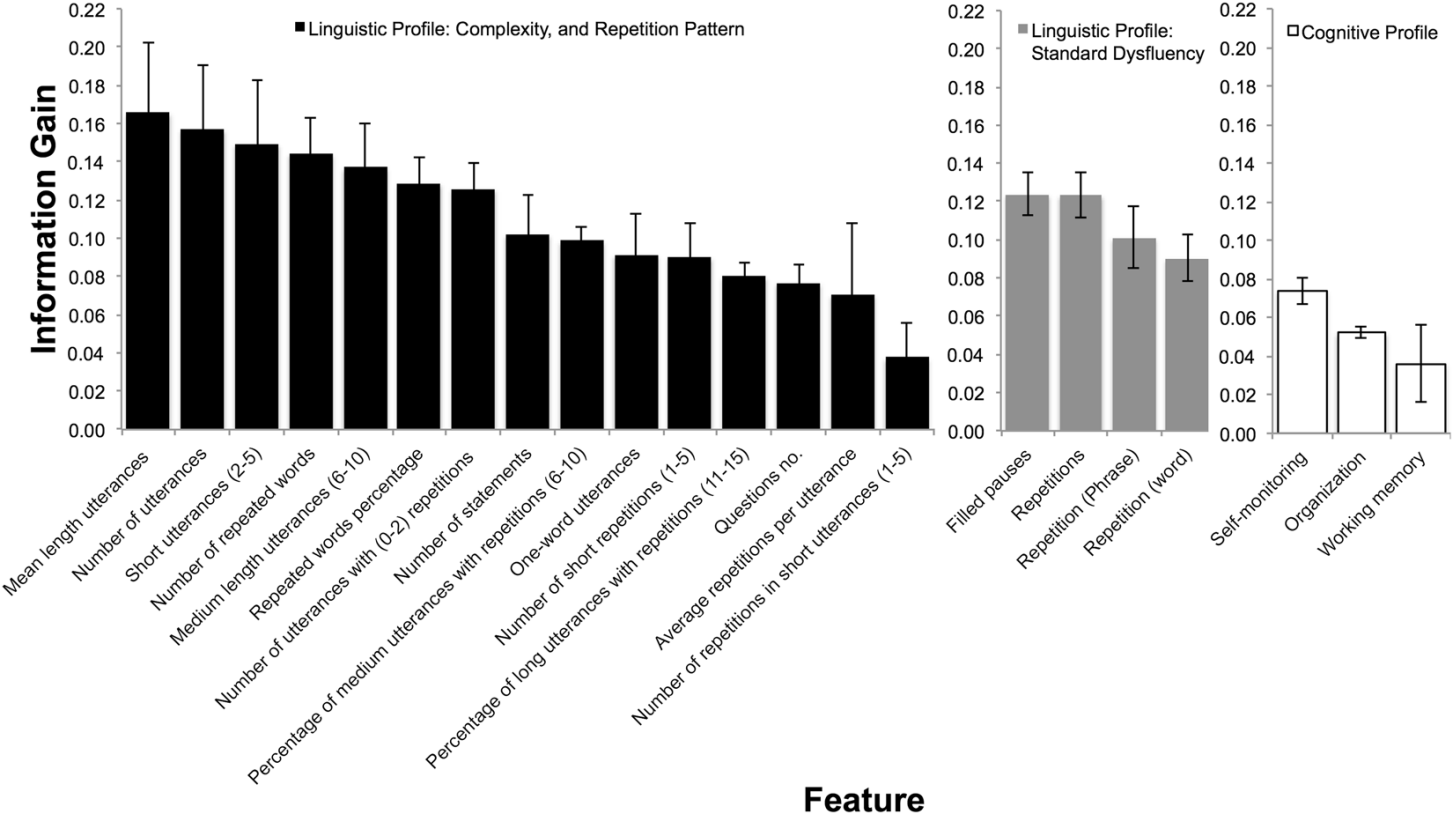
Valuable features with highly ranked information gain. Values are reported as mean ± standard deviation and presented in descending order within each type of feature. Higher value indicates higher information gain. See Table 2 for a description of the features.

Description of dysfluency features are defined as previously^11^ and cognitive features are defined as previously^32^. See Fig. 2 for information gain values.

In order to validate the reliability of the feature extraction module, we have compared the outputs with manually coded transcripts analyzed by a well-known language analysis software called the Systematic Analysis of Language Transcripts (SALT)^36^. The automated profiles were consistent with manually SALT coded profiles with more than 90 percent agreement in all the features (see Supplementary Table S3).

Figure 1A outlines the workflow for this study. After collecting the cognitive and linguistic data, our analysis starts with a feature extraction module. This module extracts linguistic features from each participant’s transcript.

Then, a comprehensive profile, containing 103 features, was generated using the combination of linguistic and cognitive features (88 linguistic features, 15 cognitive and demographic features). Next, a feature selection module was implemented to choose the most informative features based on the information gain score. A classifier was subsequently developed to determine the FX premutation status. Finally, the results of evaluation were used to determine the feasibility of improving the data collection process. A mobile app based on these results was developed to support future research.

### Informative Feature Selection

In order to create an optimized profile, we used a feature selection module based on “information gain” to define the most useful set of features for classification purposes. Information gain measures the amount of information in each feature with respect to the class prediction. In other words, information gain tells us the importance and value of a feature for discriminating between the classes (see also the Methods section)^37, 38^.

After data normalization, all of the cognitive and linguistic features were evaluated for information gain (Fig. 2 and Table 2). Linguistic features have a higher information gain compared to the cognitive features. Highly-ranked linguistic features include number of filled pauses and repetitions, as well as features related to dysfluency pattern and complexity such as number and percentage of dysfluency occurrences in utterances with different lengths. Organization of materials, the ability to evaluate performance, and self-monitoring are the cognitive variables with non-zero information gain.

### Developing a Robust Classifier

**Figure 3:**
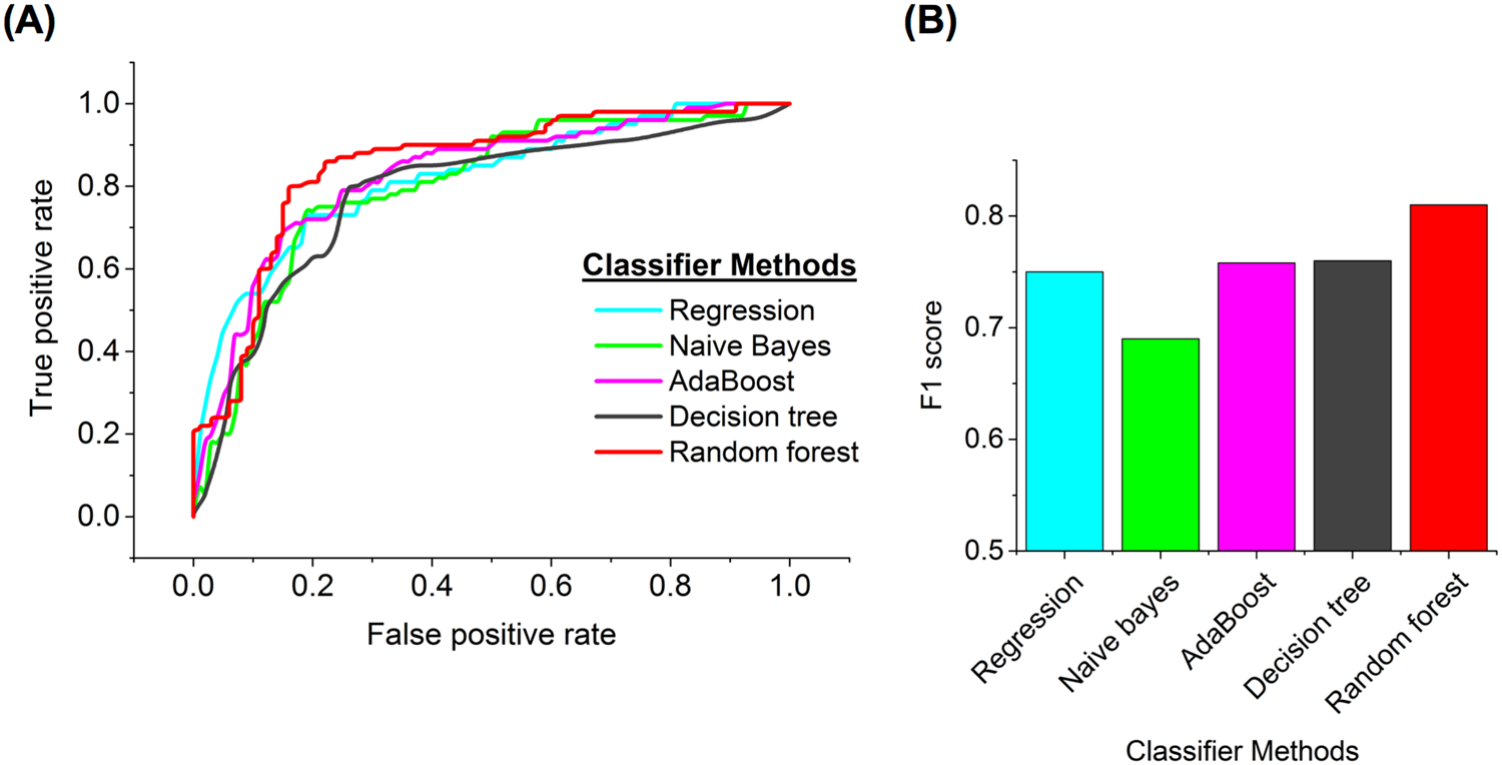
Performance of different classifiers. (**A**) Receiver operating characteristic (ROC) curves for five commonly-used classifier methods. Inputs for the classifiers utilized the full linguistic and cognitive profiles of FX premutation carriers and the comparison group. ROC curves provide a comprehensive visualization to summarize accuracy of prediction methods. The diagram shows a test’s false-positive rate (FPR), or 1 – specificity versus its sensitivity. (**B**) F1 score measures the test’s accuracy. It considers both precision and recall. As the F1 score approaches 1, the test has better accuracy. Random forest has F1 score equal to 0.81, which indicates the best performance among the tested classifiers.

We compared the performance of five commonly used classification methods for this dataset: logistic regression, Naïve Bayes, AdaBoost, Decision Trees, and Random forest^39–44^. We used 10-fold cross validation in order to train and test the model, where the dataset is randomly split into 10 subsets (folds) of equal size. The classifier is trained and tested 10 times, each time using one of the subsets for testing and the other nine for the training. The performance of classifiers for various profiles is shown in Fig. 3 and Table S7. Using all data, random forest classifiers had the best classifier performance with an F1 score of 0.81, area under the curve (AUC) of 0.84, and Matthew’s correlation coefficient (MCC) of 0.61, with decision tree being next highest with an F1 score of 0.76, AUC of 0.82, and MCC of 0.52 (Fig. 3 and Supplementary Table S7).

**Figure 4:**
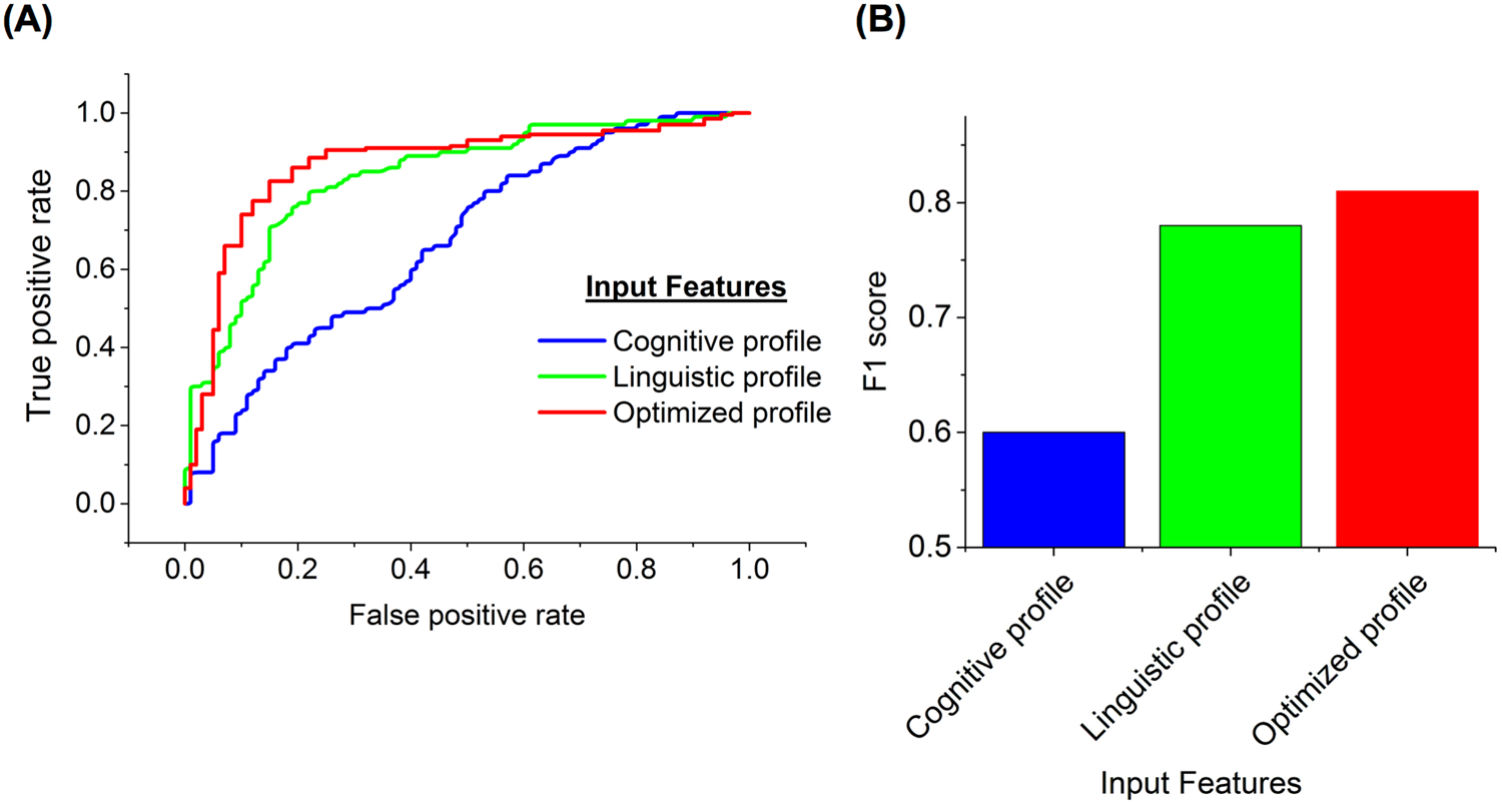
Random forest classifier performance for different sets of input features. (**A**) Receiver operating characteristic (ROC) curves for classifiers using three different profiles of FX premutation carriers and the comparison group. ROC curves provide a comprehensive visualization to summarize accuracy of prediction methods. The diagram shows a test’s false-positive rate (FPR), or 1 – specificity versus its sensitivity. Cognitive profile has the worst diagnostic utility, and our proposed profile has shown the best performance. (**B**) F1 score measures the test’s accuracy. It considers both precision and recall. As the F1 score approaches 1, the test has better accuracy. The proposed profile has F1 score equal to 0.81, which indicates the best performance among the tested profiles.

We then proceeded to develop a random forest classifier in order to evaluate the efficiency of the reduced and optimized phenotypic profiles (Fig. 4 and Supplementary Table S8). Classifiers using cognitive features only (i.e., BRIEF-A scores) showed the lowest performance (F1 score of 0.58, AUC of 0.67, MCC of 0.21). Considering all of the linguistic features, we reached higher accuracy (F1 score of 0.78, AUC of 0.83, MCC of 0.56) compared to the cognitive profile. Through our information gain analysis, we selected a limited set of features with high scores (Fig. 2) and used them for classification. The feature selection module was applied to the training data and the resulting set of features, termed the ‘optimized profile,’ was used for testing the classifier. This combination of cognitive features and linguistic profile resulted in the best performance for both FX premutation carriers and the comparison group (F1 score of 0.81, AUC of 0.85, MCC of 0.61).

In addition to 10-fold cross validation methods, we tested of our classifier using an independent set of 10 FX premutation carriers and 10 from the comparison group that were not used for training the model in the previous sections. The 10 FX premutation carriers in this set had 67, 68, 69, 70 (2), 71, 79, 83, 106 and 130 CGG repeats in *FMR1*. Using our optimized classifier, we were able to classify all FX premutation carriers correctly, with improper classification of only two participants in the comparison group, resulting in an overall F1 score of 0.91 and AUC of 0.97. This performance on an independent dataset is significantly higher than the baseline random classification (AUC of 0.5). These results provide strong evidence of the phenotypic value of linguistic and cognitive profiles to identify FX premutation carriers. However, the dataset contains FX premutation carriers with long CGG repeats, and further studies are required before using this framework to screen for FX premutation carriers with lower range of CGG repeats.

### Parsing Linguistic Features

**Figure 5:**
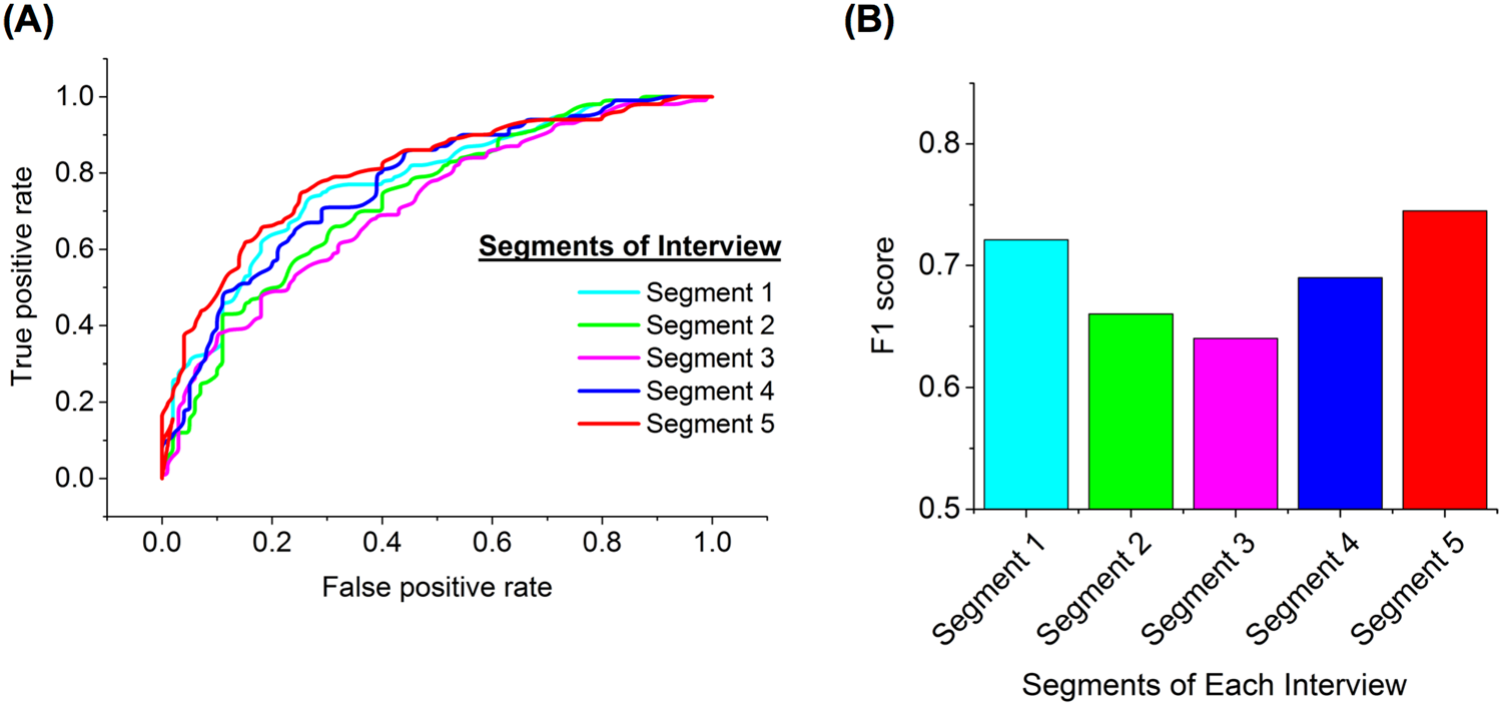
Random forest classifier performance for different segments of each interview. (**A**) ROC curves for classifiers using 5 different data profiles of FX premutation carriers and comparison group. These profiles created from a different segment of each interview, but with the same length. The first and last segments of the language samples provided the most amount of information for the classifier. The information provided in segment 3 resulted in the worst performance. (**B**) F1 score measures the test’s accuracy considering both precision and recall. The profile constructed from the last segment has a F1 score equal to 0.74, which indicates the best performance among the tested profiles from a particular segment of an interview.

To investigate the value of information provided in the different parts of the language samples, which were intended to cover five minutes of language, each sample was divided into five segments and we examined each segment and compared the results (Fig. 5, see also Supplementary Fig. S4). The first and fifth segments of interviews resulted in a significant increase in the number of dysfluencies in FX premutation carriers compared to the comparison group. While the number of filled pauses decreased over the segments in both groups (see Supplementary Table S9), FX premutation carriers had a greater number of filled pauses than the comparison group for each segment interval of the interviews (see Supplementary Text). We observed significant differences in the number of repeated words in the FX premutation carriers over different segments. However, this number was stable across the five segments of the interview for the comparison group. More repetitions were observed in the fifth segment for both groups compared to the fourth segment, and FX premutation carriers had consistently more repetitions compared to the comparison group for all parts of the interviews (see Supplementary Table S9).

### Mobile App Prototype

The optimized profile described above consists of executive function features as well linguistic dysfluency and complexity patterns collected from 5-minute language samples. This profile could be created using simple questionnaires and voice recording activity via mobile devices. Using the ResearchKit framework, we designed and created an app called “FX-PM Test” that includes a survey about our study, including a consent questionnaire, cognitive questionnaire based on BRIEF-A and an active task of voice recording for five minutes (see Supplementary Text). Our app is designed to conduct efficient experimentation with a set of easy and well-defined activities that can be performed by participants within a short period of time. The code for the prototype is discussed further in the Supplementary Text.

## Discussion

The present study investigated the cognitive and linguistic phenotypes associated with the FX premutation. We characterized the linguistic phenotypes in terms of complexity, density and dysfluency patterns. The FX premutation carriers in this study consisted of mothers of children with full mutation FXS. Therefore, they experience a high level of parental stress, which could in turn lead to more language dysfluencies rather than the consequences of the FX premutation. For this reason, we included mothers of children with autism spectrum disorder as a comparison group, due to the fact that both groups of women share a similar parenting experience and similar levels of parental stress. We also matched both groups on several important features, including age and education level.

Using the whole linguistic profile, we demonstrated that the density of language and dysfluency occurrences must be considered as important features. Dysfluency features alone may not capture the full range of differences in FX premutation carriers versus the comparison group. Therefore, by using other linguistic features as well as cognitive features, we sought to improve the accuracy of the classifier. By filtering the low ranked features and extracting the most valuable and informative features, we have built a data-driven classifier with the highest precision and recall (F1 score of 0.81, Fig. 4, Supplementary Table S8). This parsimonious approach avoids using the whole profile and therefore reduces the numbers of parameters to estimate. The optimized profile for FX premutation carriers provides an efficient tool that can be used in future research to track and monitor linguistic and cognitive phenotypic changes.

A deeper look at the linguistic profiles indicated a significant difference between FX premutation carriers and the comparison group. These differences not only included the number of dysfluencies but also comprised the pattern of dysfluency occurrences and density of language (see Supplementary Fig. S2). FX premutation carriers had more dysfluent language patterns and tended to use simpler and shorter utterances than the comparison group. In general, we found that the majority of the dysfluencies occurred in short utterances (5 words or less per utterance, see Supplementary Table S4). This reflects the distribution of utterances in our transcripts overall: there were more short utterances than long ones (10 or more words per utterance). The number of utterances with one or more repetitions was higher for FX premutation carriers than the comparison group (see Supplementary Fig. S2), suggesting that mothers of individuals with ASD are better able to plan and successfully execute their language compared to the FX premutation carriers.

In our examination of the executive function profile measured by the BRIEF-A, we did not find significant group differences in overall executive functioning, or in the working memory subdomain (see Supplementary Fig. S3). However, we found organization of materials, the ability to evaluate performance, and self-monitoring as important inputs for the classifier (Fig. 2), with poorer scores for FX premutation carriers than the comparison group. Neither cognitive deficits nor linguistic difficulties alone provided a strong a classifier, but when we considered them together, the probability of identifying impaired functioning increased significantly (Fig. 4).

The beginning and end of the language samples provided the most informative features for discriminating FX premutation carriers from the comparison group (Fig. 5). FX premutation carriers have more difficulties successfully planning and organizing their language output, which was reflected in significantly higher numbers of dysfluencies, and overall shorter, less complex statements specifically in the first and last segments of interviews. In sections demarcated by segments 2–4 of the interviews, we observed a more similar pattern of language in both groups (Fig. 5). We observed the most differences in number of dysfluency patterns and filled pauses in the last part of language sample. The full five-minute samples were used to train and test the classifier, and contained the highest amount of information in the samples (see Supplementary Fig. S4). We created a mobile app to collect the data relevant for this analysis. If used to facilitate population screening, the data collected from the mobile app could constitute a large training data set for our automated approach. Because random forest classifiers do not overfit to the training data set and have high generalization power^44^, we anticipate that the performance of our classifier in screening to improve with population size.

Examiners and researchers face some difficulties as well in scaling up studies like this one. Lack of staff and the required training for projects involving large samples with multiple data points, time constraints and funding problems are also major barriers to phenotypic research. In the linguistic and behavioral studies, in addition to the need for highly trained examiners to perform interviews and data collection, research labs must hire and train personnel to execute the language transcription and coding, which is extremely expensive in terms of both time and resources. Developing further experiments with the proposed mobile app, which eliminates the necessity to visit a data collection site or participate in a data collection telephone interview, will facilitate investigating a larger and more diverse pool of participants as well as providing the possibility of using resources and personnel more efficiently.

The current study is limited to female FX premutation carriers with children who have the full mutation of FXS. All these cases are clinically ascertained females and usually have greater than 70 CGG repeats (mean CGG length was 98). Although women with larger CGG repeats have higher risk of having a child with Fragile X syndrome, FX premutation carriers with lower range CGG repeats should be included in future studies to more fully represent the full spectrum of FX premutation carrier cases^23^. Future research should investigate FX premutation carriers whose children do not have this diagnosis or other disabilities.

The comparison group of this study is restricted to mothers of adolescents and adults with a different disability, which should be expanded in the future studies. In particular, comparison group women from the general population or other clinical groups (such as women at risk for other neurocognitive disorders like Parkinson’s disease and Alzheimer’s disease) would provide informative data. In addition, a larger number of participants with more diversity in race, age, education and geographic location could examine with this approach in future studies. Also, men with the FX premutation have not been studied in this framework. Future research is needed where the framework is tested with larger number of controls than premutation carriers, prior to using this approach in population screening. Lastly, we used a five-minute language sample and found that the end of the sample was the most informative part of the language sample. It is possible that longer language samples could improve the outcome of classifier.

Finally, impairments in language occur in a variety of clinical syndromes, including individuals with neurodegenerative diseases such Parkinson’s disease^20^, and Alzheimer’s disease^21^. Thus, the developed system can potentially be customized to further study these conditions as well as expand data collection approaches in neurocognitive studies and computational phenomics.

The collection of language samples that can predict genotypes raises ethical issues that warrant consideration, as explored recently^45–48^. Currently, although it is technically possible for each person’s genome to be fully known, a number of barriers have limited population screening for all but a small number of life-threatening conditions (e.g., those included in newborn screening). Barriers to population screening include cost, feasibility of processing a very large number of samples in the laboratory, and limitations in the number of trained genetic counselors and others to interpret the results. Population screening in the US for females with our classifier is estimated to have a positive predictive value of 2.69% (vs. 0.662% or 1/151 for general FX premutation prevalence among females, see Supplementary Table S11), leading to a four-fold decrease in the resources needed to detect FX premutation status with follow-on genetic testing. The estimates of costs savings for male FX premutation carriers depend on further studies that need to establish the ability of the language sample to correctly classify them versus controls. Furthermore, there is controversy about whether the premutation of *FMR1* constitutes a condition of concern or simply a gene variant. Studies like the present one, as well as studies that we and others we have conducted^4^, suggest that there may be cognitive and linguistic phenotypic consequences of the FX premutation. However, additional research is needed, and the use of an automated framework to process language samples has the potential to advance this research, while reducing costs and barriers to participation.

In conclusion, the present study advances our understanding of the cognitive and linguistic phenotype that distinguishes FX premutation carrier mothers of individuals with FXS from their counterparts who are mothers of individuals with ASD in three ways: (1) it identifies features that can identify FX premutation carriers efficiently without the need for clinical coding of the language; (2) it suggests that the beginning and ending of a brief language sample may elicit greater levels of dysfluency in FX premutation carriers (and hence may be a greater linguistic and cognitive challenge) than the middle sections; and (3) it reveals the value of spontaneous language as a source of important information, possibly of greater value that clinically-validated measures.

## Materials and Methods

All the modules for this project were developed in python 2.7.10 and R 3.2.2. The Origin (OriginLab, Northampton, MA) was used to create the graphs. Our custom code is available upon request.

### Language sample collection and feature extraction

The participants were asked to speak about their son or daughter for five minutes without interruption. The specific instructions are shown Fig. 1. These interviews were audio recorded and transcribed. All files were checked and finalized by a second transcriber^11^.

### Linguistic profiling

Features extracted from the language samples were compiled into a single linguistic profile for each participant. We defined various types of dysfluency features: these features are published conventions in clinical populations including FX premutation carriers, traumatic brain injury, conduct disorder, and neurodegenerative diseases^11^. The list and definition of features are provided in Supplementary Table S1.

### Cognitive profiling

Executive function consists of a set of interrelated high order skills and refers to cognitive processes including attentional control, inhibitory control, working memory, problem solving and planning^33^. Therefore, they are the mental processes that enable an individual to set a goal, develop and organize plans and monitor her/his success.

Deficits in executive functioning have been reported in FX premutation carriers^4^. We used the Behavior Rating Inventory of Executive Function Adult Version (BRIEF-A)^32–35^ to measure and assess mental executive function and self-regulation skills in our sample of FX premutation carriers and the comparison group.

The BRIEF-A can be used with adults aged 18–90. It yields an overall score (Global Executive Composite, GEC), which is a composite of two indexes (Behavioral Regulation Index: BRI, and the Metacognitive Index: MI). The BRI is a combination of four measures (Inhibit, Shift, Emotional Control, and Self-Monitor), and the MI is combined of five scales (Initiate, Working Memory, Plan/Organize, Task Monitor, and Organization of Materials). It also includes three validity measures: Negativity, Infrequency, and Inconsistency^30–33^. The definition of features is listed in Supplementary Table S5.

### Feature selection

Dimensionality is a challenging issue in large datasets that should be addressed in model construction by appropriate feature selection strategy. Feature selection can improve the accuracy of machine-learning algorithms by avoiding over fitting to the training samples and improving the generalization of the model^49–51^. Feature selection is a process, in which a subset of relevant attributes is chosen selectively by eliminating irrelevant and redundant features. This technique improves the performance and speed of the learning process in model construction. Plus, lowering the number of dimensions reduces the computational cost.

We have used *information gain*, a correlation-based feature selection strategy, to form the optimized profile. Correlation based approaches evaluate and rank a subset of features rather than individual features. These methods calculate the correlation between features themselves and also correlation between features and the class^37, 52^. Information gain, which is calculated in Eq. 1, measures the amount of information in each feature regarding to the class prediction. It evaluates the significance of each feature with respect to the class.

1 $${\rm{Information}}\,{\rm{Gain}}({\rm{Class}},{\rm{Feature}})={\rm{H}}({\rm{Class}})-{\rm{H}}({\rm{Class}}|{\rm{Feature}})$$

In this equation, H is information entropy defined by Eq. 2.

2 $$H(X)={E}_{x}[I(x)]=-{\sum }_{x\in X}p(x)\mathrm{log}\,p(x)$$

where p(x) is the probability of x and E(x) shows the expected value. Information entropy indicates the uncertainty associated with a random feature. In other words, information gain measures the expected reduction in uncertainty using a specific feature to predict a class^37, 38^.

### Classifiers

We compared the performance of five commonly-used classification methods for this dataset (Fig. 4). A random forest classifier is among the best performing classifiers. In this approach, a randomized tree-building algorithm, using the training set, grows many decision trees. Each tree predicts the class for the input sample independently. After a large number of trees are generated, they vote for the most popular class^42, 44^. Random sets of features were used to construct each tree, which makes the random forest classifier an effective prediction tool. This classifier does not over fit due to the law of large numbers, stating that the sample average converges in probability towards the expected value. The random forest classifier is robust to outliers and noise, and has a low generalization error along with excellent predictive performance^44, 53^.

For testing of the optimized classifier with an independent sample set, we used 20 samples from “Family Adaptation to FXS” and “Adolescents and Adults with Autism” studies that were not used in the training of the classifier. The 10 FX premutation carriers in this set had 67, 68, 69, 70 (2), 71, 79, 83, 106 and 130 CGG repeats in *FMR1*. The 10 participants in the comparison group had no significant differences in age or education.

### Evaluation metrics

Various measures were used to evaluate the performance of each developed model. We have measured parameters including sensitivity, specificity, accuracy, F1 score and Matthews correlation coefficient (Equations 3–7)^54, 55^:

3 $$Sensitivity=\frac{TP}{TP+FN}$$

4 $$Specificity=\frac{TN}{TN+FP}$$

5 $$Accuracy=\frac{TP+TN}{TP+FP+TN+FN}$$

6 $${MCC}=\frac{(TP\ast TN)-(FP\ast FN)}{\sqrt{[TP+FP][TP+FN][TN+FP][TN+FN]}}$$

7 $$F1=\frac{2TP}{2TP+FN+FP}$$

where TP, TN, FP and FN are True Positives, True Negative, False Positives and False Negatives, respectively. We have also used Area Under Curve (AUC) values using Receiver Operating Curve (ROC) plots. ROC curves provide a comprehensive visualization to summarize accuracy of prediction methods. The diagram shows a test’s false-positive rate (FPR), or 1 – specificity versus its sensitivity. The classifiers, models and mobile app are available upon request.

### Ethics Approval

All work with human subjects was carried out in accordance with institutional, national, and international guidelines and approved by the institutional review board at the University of Wisconsin-Madison. Written informed consent was obtained prior to testing. Additional methods and discussion of results are in the Supplementary Text.

## Electronic supplementary material

Supplementary Materials
