# Supplementary material for: Automated screening for Fragile X premutation carriers based on linguistic and cognitive computational phenotypes
Source: Sci Rep. 2017 Jun 1;7:2674. doi: 10.1038/s41598-017-02682-4 (PMC5454004; doi:10.1038/s41598-017-02682-4)
Supplement: Supplementary file 1 — Supplementary Materials [file 41598_2017_2682_MOESM1_ESM.pdf]

SUPPLEMENTARY MATERIALS

Automated screening for Fragile X premutation carriers based on linguistic and cognitive computational phenotypes

Arezoo Movaghar[1,2,3], Marsha Mailick[1]*, Audra Sterling[1,4]*, Jan Greenberg[5], Krishanu Saha[1,2,3]*

[1]Waisman Center, University of Wisconsin-Madison, Madison, WI

[2]Wisconsin Institute for Discovery, University of Wisconsin-Madison, Madison, WI

[3]Department of Biomedical Engineering, University of Wisconsin-Madison, Madison, WI

[4]Department of Communication Sciences and Disorders, University of Wisconsin-Madison, Madison, WI

[5]Department of Social Work, University of Wisconsin-Madison, Madison, WI

*To whom correspondence should be addressed: ksaha@wisc.edu, marsha.mailick@wisc.edu, audra.sterling@wisc.edu

One Sentence Summary: Efficient machine learning on short language and cognitive profiles can identify individuals carrying a *FMR1* premutation.

The supplementary materials include:

Fig. S1. Traditional workflow to create linguistic profiles.
Fig. S2. Distribution of linguistic features for FX premutation carriers and the comparison group.
Fig. S3. Distribution of cognitive features for FX premutation carriers and the comparison group.
Fig. S4. Performance of random forest classifier by using different length of transcripts as the input.

Table S1. Description of some standard linguistic features that had zero information gain.
Table S2. Linguistic features for the language sample in Supplementary Text.
Table S3. Comparison of automated feature extraction module and manual SALT methods.
Table S4. Group differences in the linguistic profile between FX premutation carriers and comparison group.
Table S5. Description of cognitive features (BRIEF-A).
Table S6. Features within in-person and over-the-phone language samples of FX premutation carriers.
Table S7. Performance of different classifiers.
Table S8. Random forest classifier performance for different sets of input features.
Table S9. Group differences between linguistic features in time segment 4 and time segment 5 in FX premutation carriers.
Table S10. Mean decrease in accuracy of fitted model after dropping each variable.
Table S11. Performance metrics of random forest classifiers for females in the US population.

Supplementary Text

Language sample 18
Text processing module 18
Comparison of linguistic profiles........ 19
Live vs. phone interviews 19
Informative linguistic features 20
Informative cognitive features 21
Length of the interviews 21
Segment differences........ 22
FX test mobile app 22

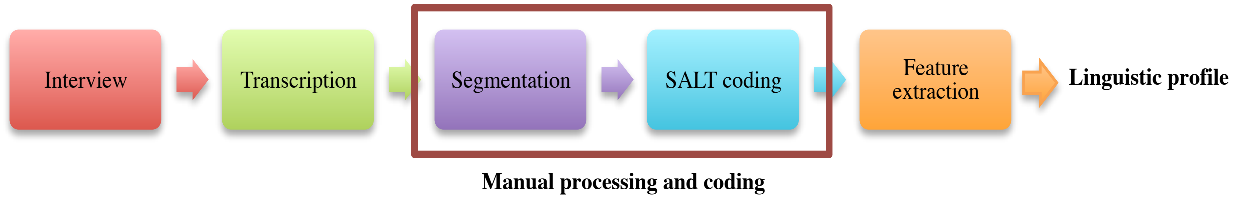

Fig. S1. Traditional workflow to create linguistic profiles. The workflow starts with data collection via phone or in-person interviews. The audio recordings were transcribed and resulting transcripts were manually segmented and SALT coded. The coded transcripts were later analyzed to create linguistic profiles. Manual coding of each transcript (red box) takes about one hour, which is time consuming, expensive and require personnel with SALT expertise. In our proposed framework we have eliminated these steps by developing an automated text-processing module to process the raw transcripts and extract linguistic features directly.

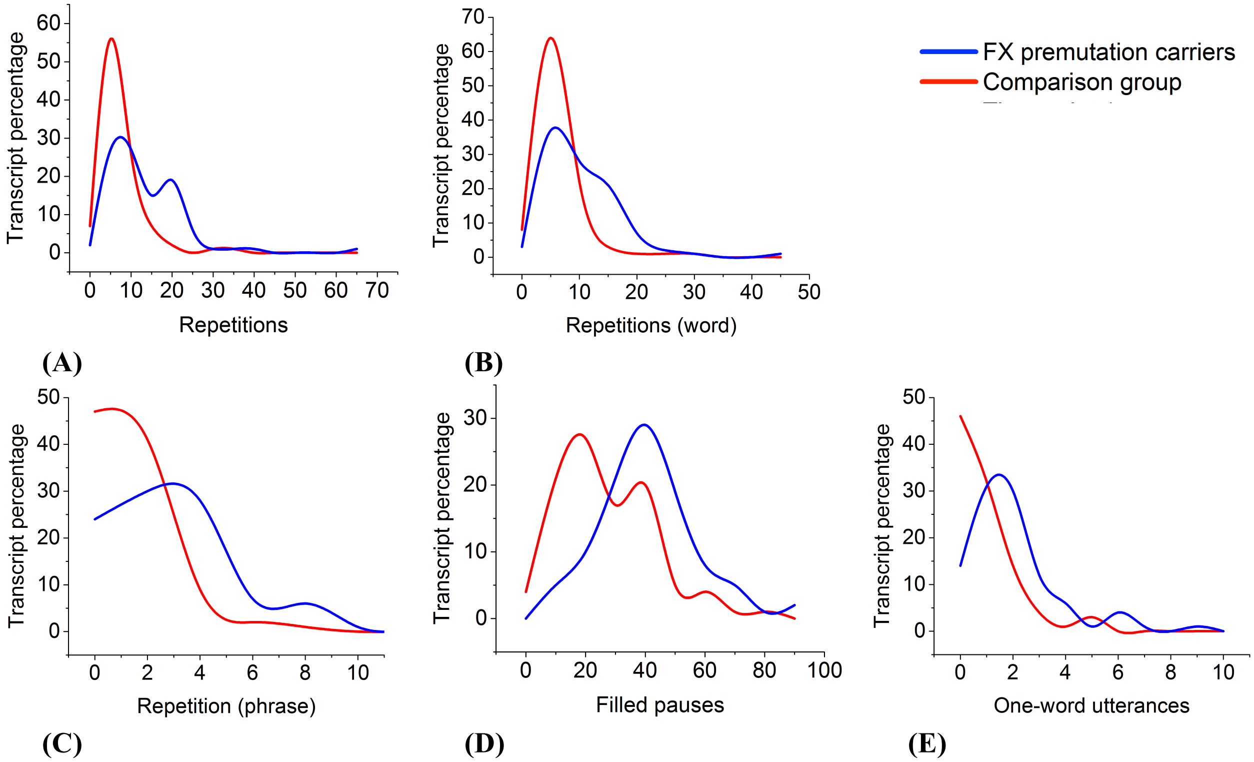

Fig. S2. Distribution of linguistic features for FX premutation carriers and the comparison group. Each graph contains the distribution of a linguistic feature in all transcripts. Dotted lines indicate data collection at two separate timepoints for each participant. These features are: **A)** Total number of repetitions in all transcripts. **B)** Number of word repetitions. **C)** Number of phrase repetitions. **D)** Number of filled pauses. **E)** One-word utterances distribution.

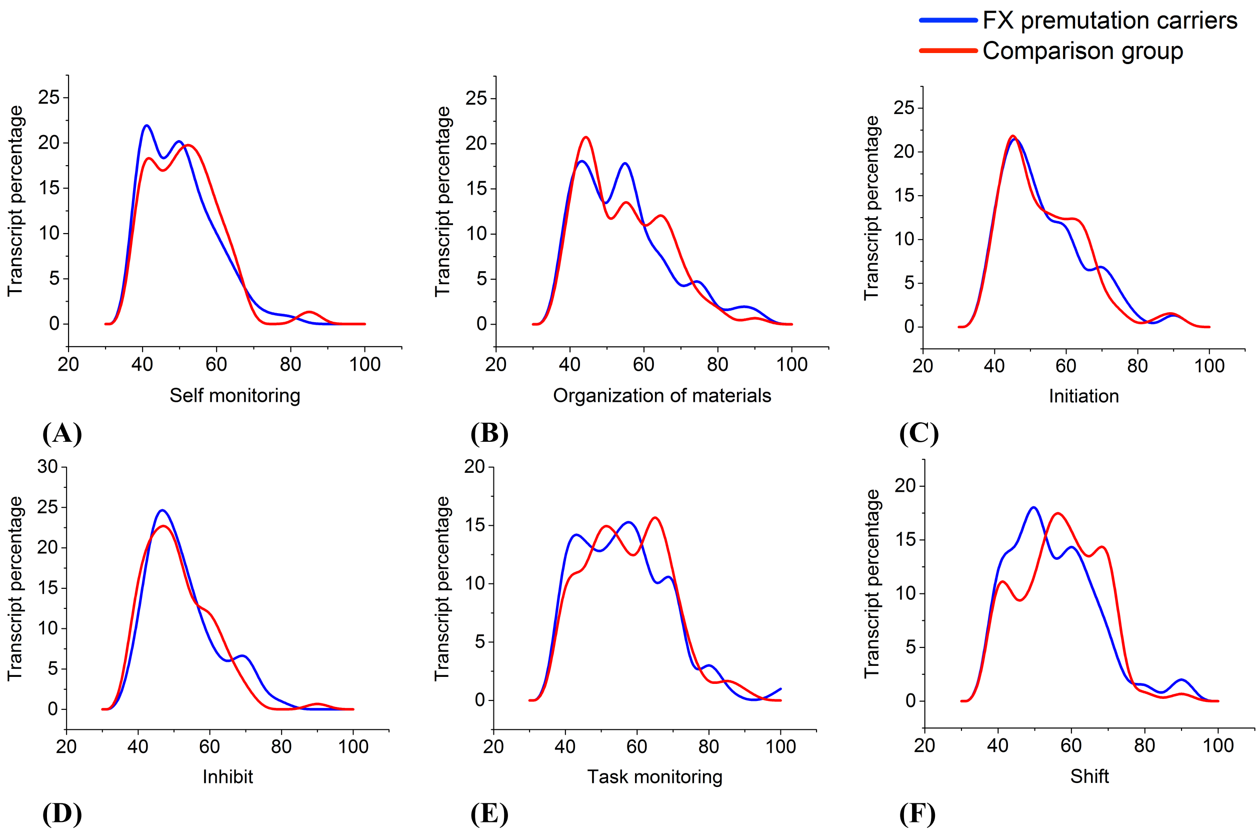

Fig. S3. Distribution of cognitive features for FX premutation carriers and the comparison group. Each diagram illustrates distribution of a cognitive feature in all transcripts. These features are: **A)** Self-monitoring. **B)** Organization of materials. **C)** Initiation. **D)** Inhibit. **E)** Task monitoring. **F)** Shift or the ability to make transition between activities.

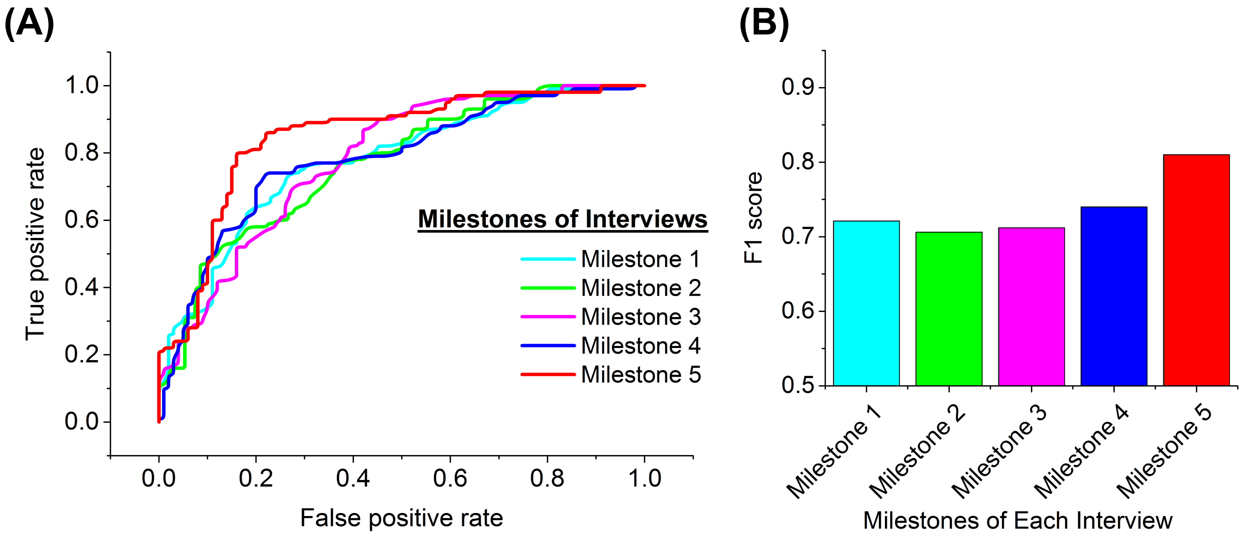

Fig. S4. Performance of random forest classifier by using different length of transcripts as the input. A) ROC curves for classifiers using 5 different data profiles of FX premutation carriers and comparison group. These profiles created from a different part of each interview. Milestone 1 indicates profile corresponding to the first segment of the interview. Milestone 2 indicates profile corresponding to the first and second segments of the interview. Milestone 3 indicates profile corresponding to the first through third segments of the interview. Milestone 4 indicates profile corresponding to the first through fourth segments of the interview. Milestone 5 indicates profile corresponding to the first through fifth segments (full length) of the interview. The full-length interviews had the best performance and provided the most amount of information for the classifier. **B)** F1 score measures the test's accuracy considering both precision and recall.

Table S1. Description of some standard linguistic features that had zero information gain. Standard definitions are employed as described in [1].

Feature	Description
**Exclamations**	Number of all the exclamations (utterances ending with “!”)
**Number of words**	Number of all the words
**Word rate**	Number of words per minute
**Utterance with dysfluency**	Number of utterances containing at least one dysfluency.
**Average word per dysfluency**	Average number of words per dysfluency (average dysfluency length)
**Number of dysfluency words**	Total number of words occurring in a dysfluency. For this study it refers to the number of repeated words.
**Long utterances**	Utterances with more than ten morphemes
**Negative utterances**	Number of utterances with negative verbs

Table S2. Linguistic features for the language sample in Supplementary Text.

Feature	Values
**Utterance No**	14
**Statement No**	13
**Questions**	1
**Mean length utterances**	8.57
**Number of words**	120
**Word rate**	24
**Total number of Repetitions**	2
**Repetition (word)**	1
**Repetition (phrase)**	1
**Filled pauses**	10
**Number of dysfluencies**	2
**Utterance with dysfluencies**	2
**Average dysfluency per utterance**	0.14
**Average word per dysfluency**	1.5
**Number of repeated words**	3

Table S3. Comparison of automated feature extraction module and manual SALT methods. Average F1 measures for fluency features between feature extraction module and SALT. F1 score values range from 0 to 1 when F1=1 indicates the exact same outputs obtained with both methods. F1=0 shows the outputs were different.

Feature	F1 score
**One word utterances**	0.95
**Total number of utterances**	0.95
**Mean length utterances**	0.93
**Filled pauses**	0.98
**Repetition (word)**	0.99
**Repetition (phrase)**	0.99
**Average repetitions per utterance**	0.97
**Number of repeated words**	0.99

Table S4. Group differences in the linguistic profile between FX premutation carriers and comparison group. Each feature was measured for each transcript for FX premutation carrier and comparison group. Features with $p<0.05$ are considered significantly different.

	FX premutation carriers		Comparison group			
**Feature**	Mean	Standard deviation	Mean	Standard deviation	*t*	*p*
**Number of utterances**	73.07	20.13	56.18	15.51	6.61	0
**Number of statements**	70.94	19.86	55.03	15.03	6.36	0
**Number of questions**	1.67	1.73	0.83	1.36	3.81	0
**One word utterances**	1.96	1.64	0.91	1.15	5.21	0
**Mean length of utterance**	10.7	2	13.11	3.34	-6.16	0
**Number of words**	761.13	176.76	707.37	179.41	2.12	0.03
**Word rate**	152.23	35.35	141.47	35.88	2.12	0.03
**Negative verbs**	3.68	2.49	3.18	2.14	1.52	0.13
**Repetition (word)**	8.4	6.65	4.37	4.56	4.97	0
**Repetition (phrase)**	2.75	3.33	1.01	1.35	4.82	0
**Total number of Repetitions**	11.15	9.04	5.38	5.45	5.44	0
**Filled Pauses**	36.49	16.17	22.36	15.69	6.24	0
**Number of repeated words**	14.48	12.17	6.61	6.68	5.64	0
**Repetitions and filled pauses**	47.64	20.11	27.74	18.35	7.27	0
**Repeated words percentage**	1.87	1.49	0.92	0.83	5.56	0
**Average repetition per utterance**	0.15	0.13	0.1	0.11	3.21	0
**Average words per repetition**	1.26	0.33	1.14	0.43	2.39	0.02

Table S5. Description of cognitive features (BRIEF-A). Standard definitions are employed as described in [2–5].

Feature	Description
**Inhibit**	The Inhibit scale assesses inhibitory control and impulsivity. This can be described as the ability to resist impulses and the ability to stop one's own behavior at the appropriate time.
**Shift**	The Shift scale assesses the ability to move with ease from one situation, activity, or aspect of a problem to another as the circumstances demand. Key aspects of shifting include the ability to (a) make transitions; (b) tolerate change; (c) problem-solve flexibly; (d) switch or alternate attention; and (e) change focus from one mindset or topic to another.
**Emotional control**	The Emotional Control scale measures the impact of executive function problems on emotional expression and assesses an individual's ability to modulate or control his or her emotional responses.
**Initiation**	The Initiate scale reflects an individual's ability to begin a task or activity and to independently generate ideas, responses, or problem-solving strategies.
**Working memory**	The Working Memory scale measures "on-line representational memory;" that is, the capacity to hold information in mind for the purpose of completing a task, encoding information, or generating goals, plans, and sequential steps to achieving goals. Working memory is essential to carry out multistep activities, complete mental manipulations such as mental arithmetic, and follow complex instructions.
**Planning/Organization**	The Plan/Organize scale measures an individual's ability to manage current and future-oriented task demands. The scale consists of two components: plan and organize. The Plan component captures the ability to anticipate future events, to set goals, and to develop appropriate sequential steps ahead of time in order to carry out a task or activity. The Organize component refers to the ability to bring order to information and to appreciate main ideas or key concepts when learning or communicating information.
**Task monitoring**	The Task Monitor scale reflects the ability to keep track of one's problem-solving success or failure, and to identify and correct mistakes during behaviors.
**Self-monitoring**	The Self-Monitor scale assesses aspects of social or interpersonal awareness. It captures the degree to which an individual perceives himself as aware of the effect that his or her behavior has on others.
**Negative**	The Negativity scale measures the extent to which the respondent answered selected BRIEF-A items in an unusually negative manner.
**Infrequency**	Scores on the Infrequency scale indicate the extent to which the respondent endorsed items in an atypical fashion relative to the combined normative and clinical samples.
**Inconsistency**	Scores on the Inconsistency scale indicate the extent to which similar BRIEF-A items were endorsed in an inconsistent manner relative to the combined normative and mixed clinical/healthy adult samples.
**Organization of material**	The Organization of Materials scale measures orderliness of work, living, and storage spaces (e.g., desks, rooms).
**Global Executive Composite**	The Global Executive Composite (GEC) is an overarching summary score that incorporates all of the BRIEF-A clinical scales.
**Behavioral Regulation Index**	The Behavioral Regulation Index (BRI) captures the ability to maintain appropriate regulatory control of one's own behavior and emotional responses. This includes appropriate inhibition of thoughts and actions, flexibility in shifting problem-solving set, modulation of emotional response, and monitoring of one's actions. It is composed of the Inhibit, Shift, Emotional Control, and Self-Monitor scales.
**Metacognition Index**	The Metacognition Index (MI) reflects the individual's ability to initiate activity and generate problem-solving ideas, to sustain working memory, to plan and organize problem-solving approaches, to monitor success and failure in problem solving, and to organize one's materials and environment. It is composed of the Initiate, Working Memory, Plan/Organize, Task Monitor, and Organization of Materials scales.

Table S6. Features within in-person and over-the-phone language samples of FX premutation carriers. No significant differences (*p*>0.05) were observed between language sample features measured via phone or in-person interviews. See also Supplementary Text.

	**In person**		**Phone**		**Independent t-test**	**Dependent t-test**
**Feature**	Mean	Standard deviation	Mean	Standard deviation	*p*	*p*
**Mean length of utterance**	13.655	2.145	13.816	3.021	0.892	0.835
**Filled Pauses**	34.5	14.990	39.7	16.138	0.106	0.457
**Number of utterances**	61.3	13.241	61.7	10.176	0.940	0.926
**Repetition (word)**	6.9	5.743	7.5	4.696	0.801	0.795
**Repetition (phrase)**	1.1	1.523	1.6	1.505	0.469	0.495
**Repetitions**	9.4	8.884	10.2	5.808	0.814	0.806

Table S7. Performance of different classifiers. Random forest has F1 score equal to ~0.81, which indicates the best performance among the tested classifiers.

Classifier	Sensitivity	Specificity	Accuracy	F1 score	AUC	MCC
**Regression**	0.76	0.73	0.745	0.749	0.807	0.49
**Naïve Bayes**	0.59	0.81	0.7	0.663	0.706	0.41
**AdaBoost**	0.72	0.8	0.76	0.75	0.78	0.52
**Decision tree**	0.75	0.77	0.76	0.758	0.82	0.52
**Random forest**	0.83	0.78	0.805	0.809	0.848	0.61

Table S8. Random forest classifier performance for different sets of input features.

Feature set	Sensitivity	Specificity	Accuracy	F1 score	AUC	MCC
**Cognitive profile**	0.56	0.65	0.605	0.586	0.67	0.21
**Linguistic profile**	0.78	0.78	0.78	0.78	0.83	0.56
**Optimized profile**	0.83	0.78	0.805	0.809	0.85	0.61

Table S9. Group differences between linguistic features in time segment 4 and time segment 5 in FX premutation carriers.

	Segment 5		Segment 4			
**Feature**	Mean	Standard deviation	Mean	Standard deviation	*t*	*p*
**Number of statements**	14.48	4.81	12.39	4.84	15.16	0
**Number of repeated words**	2.41	2.31	1.86	2.07	2.08	0.04
**Total completed words**	144.13	49.38	124.94	48.01	4.76	0
**Repetitions**	1.68	1.57	1.31	1.52	2.01	0.04
**One word utterances**	0.35	0.67	0.19	0.61	2.36	0.02

Table S10. Mean decrease in accuracy of fitted model after dropping each variable. For each tree in the random forest, the prediction error rate for classification was recorded. Then the prediction error rate after permuting each feature was measured. The difference between the two was averaged over all trees, and normalized by the standard deviation of the differences [6].

Features	Mean Decrease in Accuracy
**Filled pauses**	12.029
**Total number of repetitions**	7.486
**Repetition (word)**	4.725
**Repetition (phrase)**	2.518
**Number of utterances**	9.353
**Number of statements**	7.19
**Number of questions**	3.749
**Mean length of utterances**	10.115
**One-word utterance**	8.054
**Number of repeated words**	10.488
**Repeated words percentage**	9.933
**Short utterances**	11.911
**Medium utterances**	5.322
**Average repetitions per utterance**	3.557
**Self-monitoring**	6.162
**Organization of material**	3.283
**Working memory**	5.319

Table S11. Performance metrics of random forest classifier for females in the US population. A) Number of females in the US with ("Affected") and without ("Normal") the FX premutation predicted to have positive and negative test results with the optimized random forest classifier without coding. **B)** Summary performance metrics for random forest classifier (optimized without coding) for the US population. PPV and NPV: positive and negative predictive values. FPR: false positive rate. **C)** Costs to identify 1,000 female FX premutation carriers via two approaches to screening: genetic test only or automated classifier with follow-on genetic test. Cost of genetic test is estimated to be $100 per test (polymerase chain reaction analysis of DNA from peripheral white blood cells, CPT code 81243), while the costs of the classifier are estimated to be negligible per test. Calculations use the prevalence of FX premutation carriers as 1 in every 151 females for 2014 Kaiser Family Foundation US population data (http://kff.org/other/state-indicator/distribution-by-gender/).

A) Classifier Without Coding, Females in US

		Condition		
		Affected	Normal	Total
**Test**	Positive	886,909	32,056,967	32,943,876
	Negative	181,656	128,227,868	128,409,524
	Total	1,068,565	160,284,835	161,353,400

B) Summary Metrics, Females in US

	Automated classifier
**Sensitivity**	83%
**PPV**	**2.69%**
**Specificity**	80%
**NPV**	99.85%
**Detection rate**	1: 181
**FPR**	0.198

C) Screening Costs, Females in US

	Number of Females To Screen	Total Test Cost
Identifying 1000 female FX carriers with genetic test	151,000	$15,100,000
Identifying 1000 female FX carriers with classifier + genetic test	37,145	$3,714,500
Difference	113,855	$11,385,500

SUPPLEMENTARY TEXT

Language sample

The following example demonstrates the linguistic features in a manually coded transcript. Dysfluencies including repetitions and filled pauses are shown. **Table S2** lists all the feature values for this example.

> Matthew is um sixteen. He has Fragile X Syndrome. He is uh warm, happy, cooperative, sweet, um handsome, athletic in his own way. He is a very talented artist. He is um a wonderful part of our family. I think we we have a really wonderful relationship. It is it is a little like acquiescent. Um so I guess the next piece would be his Fragile X behavior. So he is um, which I probably like over look half the time but he hand slaps and postures. He is uh cognitively very challenged. Um, he is very dependent on us um but he is really a joy. He gives back more than he takes. Um what more can I say? I think I said everything that comes to mind.

Text processing module

We have developed a text-processing module to extract language characteristics from the transcripts (**Fig. S1)**. This module is able to batch process all the samples and create a comprehensive dataset as the refined output. The resulting dataset also includes combined attributes such as total dysfluencies, verbal information flow and frequency of dysfluencies. We have also extracted utterance distributions and dysfluencies distribution.

In order to validate the feature extraction module, we have compared its output with the output of a language analysis software called the Systematic Analysis of Language Transcripts

(SALT)[7]. F1 score was calculated for each feature and each cluster. The average F1 score for each feature among all the transcripts was reported (**Table S3**). The text processing outputs are very close to the SALT outputs.

Comparison of linguistic profiles

We have developed an exploratory data analysis module to evaluate normality and variance homogeneity in FX premutation carrier and comparison group samples. Independent sample *t*-tests were used to find significantly different features in two groups. A *p*-value of less than 0.05 was established for statistical significance. A list of features, which were assessed to be significant, has been reported in **Table S4**. FX premutation carrier samples were significantly different from the comparison group in terms of dysfluency variables. A higher frequency of dysfluency patterns was observed in FX premutation carriers.

When comparing the group, we found that filled pauses occurred in FX premutation carriers (average ~36) more frequently than the comparison group (average ~22). Overall, more dysfluency features occur in FX premutation carriers (11.15) than in the comparison group (5.38). On average about 52% of utterances in FX premutation carriers had at least one repetition or filled pauses, while the average for the comparison group was 45%.

In-person vs. phone interviews

The measures used in this study were collected either in-person or over the phone. We have investigated the features obtained from various interview methods in order to measure reliability of data our sample.

Five-minute language samples were obtained from the same group of FX premutation carriers in two separate interviews. Each participant completed both an in person and over the

phone interview. The order of performing live and over the phone interviews was varied so some of the participants completed the in person interviews first and then were called over the phone, whereas others first completed the phone first, followed by the live interviews.

We have performed both dependent and independent two samples *t*-tests to compare the differences in linguistic features. The results are listed in **Table S6**. According to the results there are no significant differences between features measured over the phone and the ones obtained in live interviews. This method can therefore be reliably used in either context resulting in the same pattern of results.

Informative linguistic features

The results from the feature selection module indicate that many linguistic features are informative, and hence we analyzed the highly-ranked features for unusual patterns and significant group differences. **Fig. S2** shows a selected set of these features to demonstrate the differences between the two groups.

According to **Fig. S2A** the FX premutation carriers had significantly ($p<0.0001$) more repetitions overall compared to the comparison group. We observed an average of 11.15 repetitions per transcript in FX premutation carriers and about 5.38 repetitions in comparison group.

In terms of the specific type of repetitions, the distribution of word repetitions in FX premutation carriers and the comparison group showed significant differences, using independent samples *t*-tests ($p<0.01$, Fig. S2B). Similar to the word repetitions, distribution of phrase repetitions for the comparison group and FX premutation carriers were significantly different ($p<0.01$, Fig. S2C).

On average, FX premutation carriers' transcripts contained more than 36 filled pauses, while the comparison group's transcripts contained about 22 filled pauses (**Fig. S2D**). FX premutation carriers used more one-word utterances than the comparison group (**Fig. S2E**), and generally, the FX premutation carriers tend to use more short utterances (less than 5 words per utterance) than the comparison group (p<0.01).

Informative cognitive features

Fig. S3 shows a selected set of cognitive features with high information values. While several studies have reported deficits in executive function skills in FX premutation carriers, we did not find significant differences between FX premutation carriers and the comparison group on the distribution of individual BRIEF-A features (p >0.05). However, some features with a high information value, such as ability to self-monitor (Information gain~0.073) organization of materials (Information Gain~0.051), and working memory (Information Gain~0.035) are important for developing robust classifiers as described below.

Length of the interviews

The interviews were analyzed in 5 milestones and the classifier performance was evaluated for each data profile. The transcript was divided into 5 segments based on the number of utterances. Milestone 1 contains 20 percent of the transcript; milestone 2 includes 40 percent and so on. The result of this analysis is shown in **Fig S4**. Longer interviews provide more reliable data.

Segment differences

The results from parsing linguistic features suggest the last part of interviews contain valuable information in order to discriminate the FX premutation carriers from comparison group. **Table S9** shows the linguistic features, which are significantly different between milestones 4 and 5 in FX premutation carriers. More repeated words were observed in the last segment of the interviews.

'FX-PM Test' mobile app

We created a demo version of a FX-PM Test mobile app. The app is a data collection platform developed using Apple's ResearchKit library. FX-PM Test provides an interactive interface to guide participants through different levels of enrollment. The process begins with introduction of the research study and its impact of public health. Prior to joining the study, participants learn about the research question, required data and data gathering methodology as well as necessary time commitment and privacy policies. Each participant voluntarily signs a digital informed consent confirming his/her interest to being involved in the study. In each data collection session, users are asked to answer a few simple questions, which will be used to develop a cognitive profile by assessing participant's executive functioning ability (i.e., memory and organization of material). The session will end with users recording their voice in respond to a given question for five minutes. The code and app are available upon request. Screenshots of the examples of activities and data collection steps are available in http://sahalab.bme.wisc.edu/FXTest/.

REFERENCES

1. Sterling, A. M., Mailick, M., Greenberg, J., Warren, S. F. & Brady, N. Language dysfluencies in females with the FMR1 premutation. *Brain Cogn.* **82,** 84–89 (2013).
2. Bourgeois, J. A. *et al.* A Review of Fragile X Premutation Disorders: Expanding the Psychiatric Perspective. *J. Clin. Psychiatry* e1–e11 (2009). doi:10.4088/JCP.08m04476
3. Roth, R. M., Isquith, P. K. & Gioia, G. A. Behavior rating inventory of executive function-adult version (BRIEF-A). *Lutz FL Psychol. Assess. Resour.* (2005).
4. Chan, R., Shum, D., Toulopoulou, T. & Chen, E. Assessment of executive functions: Review of instruments and identification of critical issues. *Arch. Clin. Neuropsychol.* **23,** 201–216 (2008).
5. Roth, R. M., Lance, C. E., Isquith, P. K., Fischer, A. S. & Giancola, P. R. Confirmatory Factor Analysis of the Behavior Rating Inventory of Executive Function-Adult Version in Healthy Adults and Application to Attention-Deficit/Hyperactivity Disorder. *Arch. Clin. Neuropsychol.* **28,** 425–434 (2013).
6. Breiman, L., Cutler, A., Liaw, A. & Wiener, M. Breiman and Cutler's Random Forests for Classification and Regression. (2015).
7. Miller, J. F., Chapman, R. S., Harry A. Waisman Center on Mental Retardation and Human Development. & Language Analysis Laboratory. SALT systematic analysis of language transcripts. (1984).
